# Cytosine methylation of tRNA-Asp by DNMT2 has a role in translation of proteins containing poly-Asp sequences

**DOI:** 10.1038/celldisc.2015.10

**Published:** 2015-06-09

**Authors:** Raghuvaran Shanmugam, Jacob Fierer, Steffen Kaiser, Mark Helm, Tomasz P Jurkowski, Albert Jeltsch

**Affiliations:** 1 Institute of Biochemistry, Stuttgart University, Faculty of Chemistry, Stuttgart, Germany; 2 MoLife Program, School of Engineering and Science, Jacobs University Bremen, Bremen, Germany; 3 Institute of Pharmacy and Biochemistry, Faculty of Chemistry, Pharmaceutical Sciences and Geoscience, Johannes Gutenberg-Universität Mainz, Mainz, Germany

**Keywords:** Dnmt2, tRNA methylation, aminoacylation, regulation of translation, Asp-rich proteins

## Abstract

The Dnmt2 RNA methyltransferase catalyses the methylation of C38 in the anticodon loop of tRNA-Asp, but the molecular role of this methylation is unknown. Here, we report that mouse aspartyl-tRNA synthetase shows a four to fivefold preference for C38-methylated tRNA-Asp. Consistently, a 30% reduced charging level of tRNA-Asp was observed in Dnmt2 knockout (KO) murine embryonic fibroblast cells. Gene expression analysis with fluorescent reporter proteins fused to an N-terminal poly-Asp sequence showed that protein synthesis of poly-Asp-tagged reporter proteins was reduced in Dnmt2 KO cells as well. The same effect was observed with endogenous proteins containing poly-Asp sequences, indicating that Dnmt2-mediated C38 methylation of tRNA-Asp regulates the translation of proteins containing poly-Asp sequences. Gene ontology searches for proteins containing poly-Asp sequences in the human proteome showed that a significant number of these proteins have roles in transcriptional regulation and gene expression. Hence, the Dnmt2-mediated methylation of tRNA-Asp exhibits a post-transcriptional regulatory role by controlling the synthesis of a group of target proteins containing poly-Asp sequences.

## Introduction

RNA methylation occurs in all types of RNA, including ribosomal RNA, transfer RNA (tRNA), messenger RNA and small RNA ubiquitously in both prokaryotes and eukaryotes [1–5]. In particular, tRNAs are extensively modified in all organisms and contain a variety of different modifications, including methylation, pseudouridylation, thiouridylation, and addition of isopentenyl groups. Despite their systematic identification, the molecular function of many of these modifications is not well understood. Methylation of tRNAs is introduced by tRNA methyltransferases that are specific towards particular tRNAs and target sites. Different types of tRNA methyltransferases have been identified based on their fold and reaction mechanism [2]. Dnmt2, also known as tRNA-aspartic acid methyltransferase 1 (Trdmt1), is a highly conserved cytosine-C5 methyltransferase that introduces the C38 methylation of tRNA^Asp^ in many species, including lower eukaryotes, plants, insects, and humans [6–12]. Interestingly, Dnmt2 resembles DNA methyltransferases in structure and catalytic mechanism [8] but it was shown in 2006 to function as an RNA methyltransferase, which methylates C38 of tRNA^Asp^ [7, 8]. Later additional substrates were identified including tRNA^Gly^ and tRNA^Val^ in human and Drosophila [5, 9, 13] and tRNA^Glu^ in *Schizosaccharomyces pombe*, Dictyostelium and Geobacter [11, 12, 14]. The strong conservation of Dnmt2 homologues in the eukaryotic kingdom [6, 15] implies a very important function of these enzymes in maintaining cellular homeostasis. Dnmt2 expression is tissue specific in humans [16,  17] and mice [18], suggesting a dynamic role of Dnmt2 signalling in mammalian cells. Along the same line, Dnmt2 was found consistently upregulated in hundreds of tumour samples listed in the COSMIC database [19]. Dynamic physiological roles of Dnmt2 have also been observed in model animals. Although Dnmt2 knockout (KO) mice lack strong phenotypes [7, 20], Dnmt2 KO flies exhibit an increased sensitivity to oxidative stress and heat shock due to the lack of C38 methylation in tRNA^Asp^ that leads to the fragmentation of this tRNA [9]. Reduced tRNA stability after loss of Dnmt2 has also been observed in mouse cells [13]. The tRNA fragments have been shown to influence the antiviral defence in Drosophila and to influence Dicer-2 processing of small interfering RNAs [21, 22]. Similar effects were observed under heat stress in flies where Dnmt2 relocalized to stress granules, leading to a reduced cellular Dnmt2 activity [9]. Still, the exact molecular connection between the methylation of C38 in tRNA^Asp^ and a reduced stress tolerance is not known.

Here, we aimed to characterise the physiological role of the dynamic Dnmt2-mediated C38 methylation of tRNAs. Given the essential role of tRNAs in protein synthesis, we investigated the influence of Dnmt2-catalysed tRNA methylation on this process. As the addition of methyl groups often has a role in modulating the specificity of protein–nucleic acid interactions, we investigated the role of C38 methylation in the charging of tRNAs. In fact, C38 has been identified as an identity element of aspartyl-tRNA synthetase (AspRS) in bacteria, archea, and eukaryotes [23]. As the C38 bases of tRNA^Gly^ and tRNA^Val^ are not important for the interaction of these tRNAs with their cognate aminoacyl-tRNA synthetase [23], we focused on AspRS and show that AspRS activity is stimulated by C38 methylation and that loss of this mark decreases the level of tRNA^Asp^ charging. Using a fluorescent reporter system and endogenous proteins containing poly-Asp sequences, we show that the loss of Dnmt2 leads to reduced synthesis of poly-Asp-containing proteins. We show the enrichment of poly-Asp proteins in genes with regulatory roles, and based on our findings we propose that Dnmt2 has a post-transcriptional regulatory role in global modulation of the expression of these proteins. This novel translational regulation pathway could participate in the Dnmt2-mediated stress response.

## Results

### Cytosine-38 methylation of tRNA^Asp^ increases the rate of its aminoacylation

**Figure 1:**
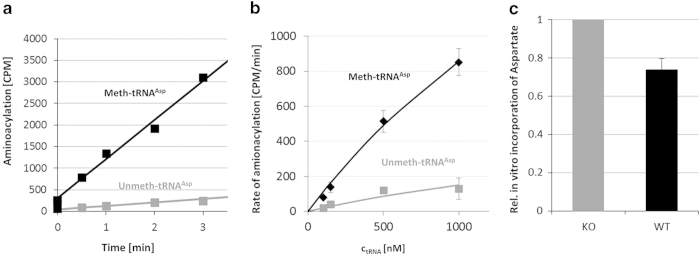
Aminoacylation of C38-methylated and unmethylated tRNA^Asp^. (**a**) Example kinetics of aminoacylation of C38-methylated mouse tRNA^Asp^ (black) and unmethylated mouse tRNA^Asp^ (grey) with the AspRS enzyme using 1 μm of transfer RNA (tRNA). (**b**) Aminoacylation rates of C38-methylated (black) or unmethylated (grey) mouse-tRNA^Asp^ determined at different concentrations of tRNA. The line shows a fit of the rates to a Michaelis–Menten model indicating a 5.5-fold increase in *V*_max_/*K*_m_. (**c**) Aminoacylation level of tRNA^Asp^ from wild-type and Dnmt2 knockout (KO) cells. The incorporation of labelled aspartate in the *in vitro* aminoacylation reactions was higher with the tRNA isolated from Dnmt2 KO mouse embryonic fibroblast cells as compared with corresponding wild-type cells. This indicates that the charging level tRNA^Asp^ isolated from Dnm2 KO cells is about 30% lower. All error bars indicate the s.e. CPM, counts per minute.

Despite strong conservation and ubiquitous presence of the Dnmt2 tRNA methyltransferase, the molecular function of the C38 methylation of tRNA^Asp^ introduced by Dnmt2 remains unknown. Searching for a direct molecular function of this tRNA methylation, we have focused on its potential role in modulating the interaction with the aspartyl-tRNA synthetase (AspRS), because modifications in the anticodon loop of tRNAs have been shown to affect their charging in other cases [24–26], and C38 has been mapped as a direct identity determinant of AspRS from species in all kingdoms of life [23]. To investigate the role of C38 methylation of tRNA^Asp^ in tRNA charging, we have cloned mouse AspRS from complementary DNA and purified the recombinant enzyme from *E. coli* [27, 28] (Supplementary Figure S1a). Next, we have determined the rates of the aminoacylation of methylated tRNA^Asp^ and unmethylyated tRNA^Asp^ using an *in vitro* aminoacylation assay [29, 30]. For this, we synthesised the mouse tRNA^Asp^ in the C38-methylated and unmethylated form by splint ligation (Supplementary Figure S1b) and used it as a substrate for aminoacylation reactions in the presence of ^3^H-labelled aspartate. We observed that the aspartylation efficiency was significantly higher with methylated tRNA^Asp^ than with unmethylated tRNA^Asp^ (Figure 1a). Experiments using the tRNA at different concentrations in the range of 100–1000 nm showed a 5.5-fold increase in the *V*_max_/*K*_m_ for the C38-methylated tRNA substrate when compared with the unmethylated tRNA (Figure 1b). These results suggest that C38 methylation is needed for an efficient charging of tRNA^Asp^ by AspRS.

### The charging level of tRNA^Asp^ is reduced in Dnmt2 KO cells

Next, we studied whether the reduced aminoacylation rate of unmethylated tRNA^Asp^ leads to reduced charging levels of tRNA^Asp^ in Dnmt2 KO cells. To this end, an incorporation assay was employed. Total RNA containing tRNA^Asp^ was isolated from murine embryonic fibroblast (MEF) cells under mild acidic condition where the charging of tRNA is preserved [31, 32]. Then, the RNA was incubated with recombinant AspRS and ^3^H-labelled aspartate, and the transferred radioactivity was quantified. As aminoacylated tRNA is refractory to the *in vitro* aminoacylation, a higher incorporation of radioactively labelled asparate in this assay is indicative of a lower aminoacylation level of the specific tRNA^Asp^. A portion of each RNA preparation was deacylated by incubation at alkaline pH [33] and treated identically to serve as input correction for the amount of tRNA^Asp^ in the RNA preparations. We observed that the aminoacylation level of tRNA^Asp^ isolated from the Dnmt2 KO cells was ~30% lower when compared with tRNA^Asp^ isolated from wild-type cells (Figure 1c). This result indicates that the loss of C38 methylation in cells leads to reduced availability of charged tRNA^Asp^. The observation that the strong reduction of *in vitro* activity of the AspRS only led to a smaller reduction of charging levels *in vivo* might be explained by the presence of other modifications *in vivo*. In addition, the aminoacylation efficiency is only one factor determining the steady-state charging levels of tRNAs *in vivo*.

### Dnmt2 KO cells show a reduced expression of Asp-tagged fluorescent reporter proteins

**Figure 2:**
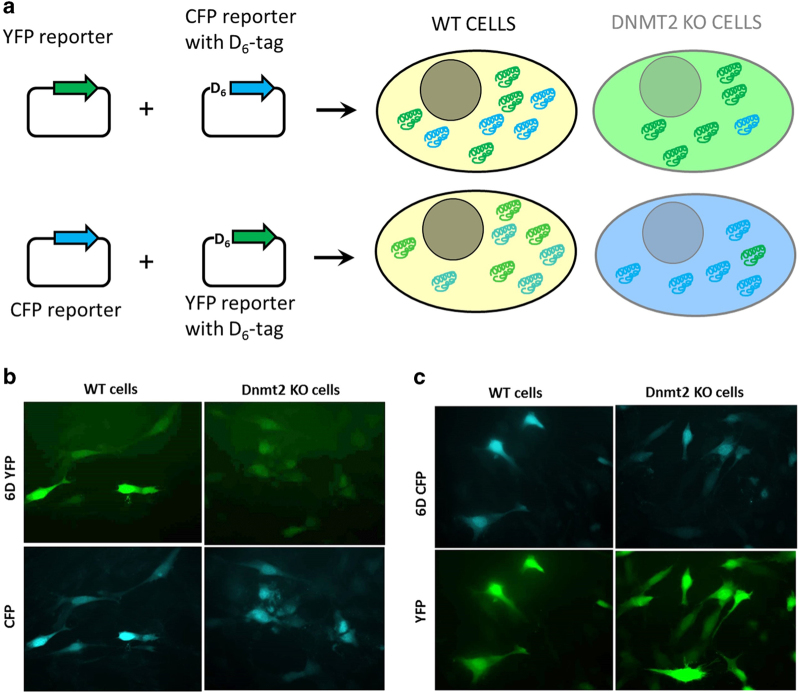
Double-reporter analysis for *in vivo* synthesis of poly-Asp-tagged proteins. (**a**) Schematic drawing of the experimental design. Wild-type (WT) and Dnmt2 knockout (KO) mouse embryonic fibroblast cells were co-transfected with a normal yellow fluorescent protein (YFP) and Asp_6_-tagged cyan fluorescent protein (CFP) or vice versa, and the expression of both proteins was quantified in individual cells. In Dnmt2 KO cells, the expression of Asp_6_- tagged protein was decreased due to reduced efficiency of translation. (**b**) Example pictures of wild-type and Dnmt2 KO cells co-transfected with 6DYFP and CFP. In Dnmt2 KO cells, the 6DYFP proteins showed a reduced synthesis. (**c**) Example pictures of wild-type and Dnmt2 KO cells co-transfected with 6DCFP and YFP. In Dnmt2 KO cells, the 6DCFP proteins showed a reduced synthesis. The images were taken 48 h after transfection and the cells were fixed by formaldehyde. See also Supplementary Figure S2.

**Figure 3:**
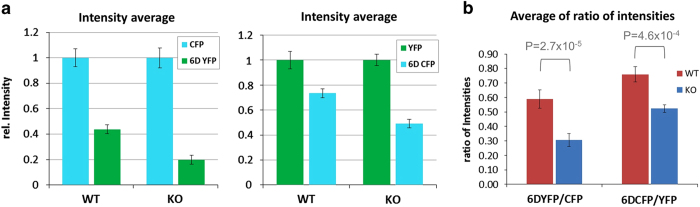
Quantitative analysis of reporter gene expression in wild-type (WT) or Dnmt2 knockout (KO) cells after co-transfection of cyan fluorescent protein (CFP) and 6DYFP or yellow fluorescent protein (YFP) and 6DCFP. Expression was analysed in ~150 cells for each experiment (Supplementary Figure S3). (**a**) Intensity averages of the YFP, 6DYFP, CFP, and 6DCFP expression in individual WT or Dnmt2 KO cells in the two co-transfection experiments. The synthesis of Asp_6_-tagged proteins was lower in all experiments, but the reduction was stronger in Dnmt2 KO cells as compared with WT cells. Intensities were normalized to the values of the untagged reporters. The error bars indicate the s.e.m. (**b**) Averages of the ratios of 6DYFP and CFP or 6DCFP and YFP expression levels in individual WT and Dnmt2 KO cells. In both experiments the KO cells showed a reduced relative expression of the Asp-tagged proteins, when compared with WT cells. The error bars indicate the s.e.m. The *P*-values were derived from a two-sided *T-*test assuming equal variance of the data sets.

Next, we wanted to investigate whether the observed reduction in the charging levels of tRNA^Asp^ leads to a decreased translational efficiency in Dnmt2 KO cells. We hypothesized that a consecutive stretch of aspartate residues present at the N-terminus of a reporter protein would allow us to challenge the efficiency of Asp incorporation in the cell. We have, therefore, constructed reporter plasmids expressing the YFP and CFP fluorescent proteins fused with an N-terminal Asp_6_-tag (6DYFP and 6DCFP) (Figure 2a). Wild-type and Dnmt2 KO MEF cells were co-transfected with two YFP and CFP reporter constructs one having an Asp-tag and the other one without. Forty-eight hours after transfection, the cells were fixed and the fluorescence intensity of YFP and CFP was measured. As shown in Figure 2b (and Supplementary Figure S2), the relative fluorescence intensity of Asp-tagged proteins was higher in wild-type cells when compared with Dnmt2 KO cells. Swapping of the reporters indicated that this effect was not due to technical artefacts. Control experiments with single transfections indicated absence of crosstalk between the YFP and CFP channels with our settings (Supplementary Figure S3). For quantitative analysis, we determined the fluorescence intensities of both reporter proteins from more than 150 individual cells for each experiment (Supplementary Figure S4). The intensity averages of the expression levels showed that the expression of the Asp-tagged reporters was lower in both co-transfection experiments (Figure 3a). However, this effect was more pronounced with Dnmt2 KO cells than with wild-type cells. Next, we calculated the ratio of 6DYFP/CFP or 6DCFP/YFP signals for individual cells such that the fluctuations of overall reporter gene expression between individual cells could be compensated. As shown in Figure 3b, the relative expression of the Asp-tagged proteins was lower in Dnmt2 KO cells than in wild-type cells, and this effect was highly significant. These data show that Dnmt2 KO cells have difficulties in synthesising Asp-tagged proteins.

### Synthesis of endogenous proteins with poly-Asp sequences is reduced in Dnmt2 KO cells

**Figure 4:**
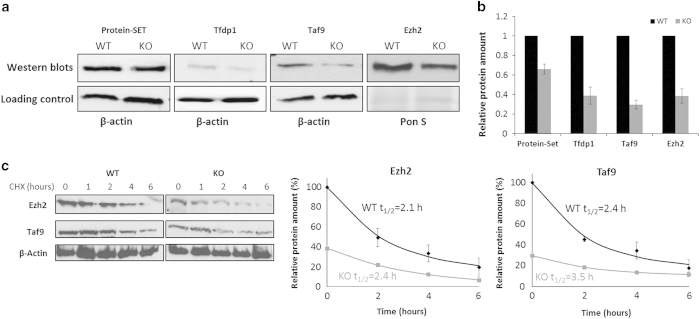
Levels of endogenous proteins with poly-Asp sequences are reduced in Dnmt2 knockout (KO) cells. (**a**) Images of western blots for specific proteins showing a difference in the amount of poly-Asp-tagged protein in Dnmt2 KO and wild-type (WT) cells (upper panel). The lower panel represents the corresponding loading controls. (**b**) Quantified data showing relative levels of specific proteins in WT (black) and Dnmt2 KO (grey) cells. The error bars show the s.e.m. from three experiments. (**c**) Degradation of the Ezh2 and Taf9 proteins after cycloheximide treatment in WT and Dnmt2 KO cells. The errors bar shows the s.e. of two repeats. The lines show a fit of the data to a single exponential decay curve. The experimental half-lives of the proteins (*t*_1/2_) as derived from these fits are indicated. The protein levels were normalized to the initial amount and background was subtracted.

After showing the reduction in the synthesis of Asp-tagged reporter proteins in Dnmt2 KO cells, we investigated whether expression of cellular proteins, which naturally contain stretches of aspartate residues, is also influenced by lack of C38 methylation in tRNA^Asp^. Using the Scansite 2.0 web server [34], we identified a total of 49 endogenous candidate proteins in the murine proteome, which contain stretches of six or more Asp residues in their sequence. From them, we have selected seven proteins that have been previously reported to be expressed in MEF cells (Supplementary Table S3). Cellular levels of these proteins were analysed in the total protein extract prepared from wild-type and Dnmt2 KO MEF cells by western blots. We observed reduced levels of protein-SET, TFDP-1, TAF9, and Ezh2 proteins in Dnmt2 KO cells compared with wild-type MEF cells (Figure 4a; Supplementary Table S1). No differences were observed with DAXX and NPM; the FGFR1 antibody failed (data not shown). The quantitative analysis showed that the levels of the transcription activation factor 9 (TAF9) protein, which contains an Asp_13_ sequence in its C-terminal region, were affected the most (Figure 4b) supporting the hypothesis that the synthesis of proteins with long Asp runs is strongly reduced in Dnmt2 KO cells. The messenger RNA expression levels of the candidate proteins were determined previously for the same cells as used here, showing that the transcript levels for the selected candidate proteins were identical in wild-type and Dnmt2 KO MEFs (Supplementary Figure S5). This result implicates that the reduced levels of these proteins are due to their reduced translation in Dnmt2 KO cells, which can be explained by the reduced level of charged tRNA^Asp^ in these cells.

### Protein degradation does not cause reduction of protein level in Dnmt2 KO cells

To test whether the reduced levels of Asp-tagged protein were due to their increased degradation in Dnmt2 KO cells, the rate of protein degradation in wild-type and Dnmt2 KO MEF cells was determined by cycloheximide chase. The wild-type and Dnmt2 KO cells were grown and treated with cycloheximide, which blocks protein synthesis. Cells were harvested at specific time points between 0 and 6 h. After cell lysis, the amounts of Taf9 and Ezh2 proteins were compared by western blot (Figure 4c), because these proteins showed the largest changes in expression levels at a high level of expression. The results indicated that both proteins were degraded with almost the same rates in wild-type and Dnmt2 KO cells, indicating that the differences in steady-state levels determined here directly reflect the reduced synthesis of Asp-tagged proteins in Dnmt2 KO cells.

## Discussion

**Figure 5:**
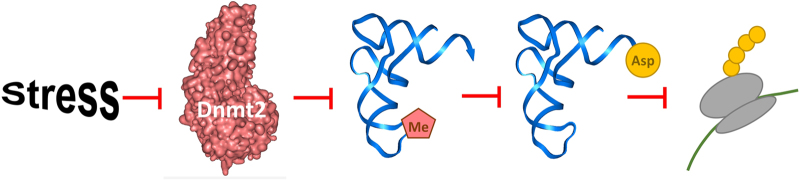
Summary of the main findings of this paper. Reduction in Dnmt2 activity reduces C38 methylation of tRNA^Asp^. This reduces the availability of charged tRNA^Asp^ and diminishes translation of Asp-rich proteins.

Modification of tRNAs has diverse roles in modulating the translational efficiency and fine tuning the cellular response to various stimuli. Here, we describe a direct molecular function of the methyl group transferred by Dnmt2 to the C38 of tRNA^Asp^ by showing that methylation supports the recognition of this tRNA by its cognate AspRS. This observation agrees with previous findings in other systems showing that tRNA methylation has important roles in charging [35, 36] and prevention of mischarging [37]. Other reported functions of tRNA methylation are in the modulation of the codon/anticodon pairing [38–40]. The loss of Dnmt2 has been shown to lead to tRNA fragmentation and reduction in the steady-state levels of tRNA [9, 13]. In the Dnmt2 KO mouse cell line also used in this work, steady-state levels of tRNA^Asp^ were reduced by 27% when compared with wild-type cells after input correction [13]. We demonstrate here that reduced Dnmt2 activity leads to an increased fraction of uncharged tRNA^Asp^ in cells (Figure 5). Together, these effects caused less efficient translation of poly-Asp-containing proteins, which we demonstrate here for the first time. The reduced translation of Asp-rich proteins was not accompanied by a general reduction in protein biosynthesis in Dnmt2 KO cells [13], indicating that it is a specific effect. The selective reduction in the translational efficiency of poly-Asp proteins observed here represents a novel mechanism of post-transcriptional regulation mediated by methylation of tRNA^Asp^. A classification of gene ontology gene functions of human proteins containing Asp-runs (Table 1) revealed that a significant proportion of these proteins have nuclear localisation, and they have roles in gene expression and transcription regulation.

Changes in the expression of Dnmt2 suggest a dynamic role of the Dnmt2-mediated C38 methylation of rRNA^Asp^, which is also supported by the recent finding of RNA hydroxymethylation activity of TET enzymes in mammalian cells [41]. Hence, the change in Dnmt2 activity under cellular stress conditions could trigger a signalling cascade in which reduced methylation of tRNA^Asp^ leads to its reduced charging, which in turn causes a reduced synthesis of proteins with poly-Asp runs, which then can change the expression of further target genes. The reduced synthesis of ‘standard’ gene regulatory proteins, thereby, could support synthesis of specific factors needed during response to cellular stress. In accordance with the mild phenotypes of Dnmt2 KO animals, the role of Dnmt2 is in the modulation of the transcriptional profile of cells leading to a fine tuning of the cellular properties to better adapt to cellular stress. Our data identify a molecular function of the C38 methylation introduced by Dnmt2 that can be directly connected to a physiological function, which lies in a system-wide novel post-transcriptional regulation mechanism. Homopolymeric stretches of amino acids other than poly-Asp are widespread in the human proteome, but their function often is not clear. On the basis of our findings, they may affect protein biosynthesis using a similar mechanism regulated by a modification of the corresponding tRNA.

Future studies are needed to address potential additional roles of the Dnmt2-mediated tRNA methylation of tRNA^Asp^ but also of tRNA^Gly^ and tRNA^Val^. Biochemical studies of ValRS and GlyRS could reveal if these enzymes respond to C38 methylation as well, although this is less likely on the basis of the known specificity determinants of tRNA^Gly^ and tRNA^Val^. Moreover, C38 methylation could help to prevent mischarging, similar as methylation of G37 in tRNA^Asp^, which has been shown to prevent mischarging by the arginyl-tRNA synthetase in yeast [37]. In addition, C38 methylation could influence the interactions of the tRNA in the ribosome. Methylation of the wobble position of the tRNA has been shown to modulate the tRNA–messenger RNA interaction and lead to altered codon preferences [39, 40]. However, the distance of the base 38 and the wobble base pair is more than 12 Å in all different ribosome structures, which makes an effect of C38 methylation on wobbling unlikely. In contrast, the distance of the base 38 to the neighbouring tRNA is less than 7 Å in some ribosome structures, which makes it possible that C38 methylation may affect kinetics of translocation or tRNA release and change the codon pair preferences [42].

## Materials and Methods

### Cloning, protein expression, and purification

The gene coding for mouse aspartyl-tRNA synthetase (AspRS) (UniProt ID: Q922B2) was amplified from complementary DNA prepared from murine embryonic fibroblast (MEF) cells using specific primers (forward 5′-GGCTAGCATGCCCAGCGCCAACGC-3′ and reverse 5′-GTGCTCGAGTTAAGGCGTGAGTCGTTTGGG-3′) and cloned into pET28a+ using *Nhe*I and *Xho*I sites. The protein was expressed in *Escherichia* *coli* BL21 (DE3) Rosetta2 cells as outlined below. Protein expression was induced with 1 mm isopropyl-β-d-thiogalactoside at an optical density (600 nm) of 0.6 and conducted at 22 °C overnight in shaking culture. After harvesting, the cells were disrupted by sonication of the cell pellet in wash buffer (50 mm HEPES pH 7.2, 500 mm NaCl, 10% glycerol, 0.1 mm dithiothreitol (DTT), 0.5 mm EDTA, 10 mm imidazole). The cleared lysate was applied onto Ni-NTA beads followed by washing steps and elution in buffer (50 mm HEPES pH 7.2, 500 mm NaCl, 10% glycerol, 0.1 mm DTT, 0.5 mm EDTA, 200 mM imidazole). The purified protein was dialysed against dialysis buffer I for 2 h (50 mm HEPES pH 7.2, 250 mm NaCl, 20% glycerol, 0.1 mm DTT) and dialysis buffer II (50 mm HEPES pH 7.2, 150 mm NaCl, 50% glycerol, 0.1 mm DTT) overnight. The cloning of 6× aspartate leader sequence into pEYFP-N1 and pECFP-N1 was achieved through ExSite PCR using the forward primer 5′-CCACCGGTCGCCACCATG-3′ and reverse primer 5′-GCTCCTCGCCCTTGCTCACGTCATCATCGTCATCGTC-3′.

### Substrate tRNA preparation

Unmethylated and C38-methylated mouse tRNA^Asp^ substrates were created by splint ligation [43, 44]. The ligation reaction was performed by annealing two synthetic RNA fragments (IBA, Göttingen, Germany) (Supplementary Table S2) corresponding in sequence to mouse-tRNA^Asp^, onto a 52-nt-long complementary oligodeoxynucleotide. Unmethylated or methylated fragments (4 nmol) were 5′-phosphorylated by incubating in KL buffer (50 mm Tris-HCL pH 7.4, 10 mm MgCl_2_) supplemented with 5 mm ATP, 5 mm DTT, and 0.75 U μl^−1^ T4 polynucleotide kinase (Life Technologies, Darmstadt, Germany) in a final volume of 150 μl in a thermomixer at 37 °C for 1 h. Afterwards, an equimolar amount of the 5′-fragment and the DNA splint dissolved in KL buffer containing 5 mm ATP and 5 mm DTT were added, leading to a final volume of 500 μl and a concentration of 8 μM of each fragment. The RNA fragments were hybridized to the DNA splint by heating to 75 °C in a thermomixer for 4 min and cooling down to room temperature within 15 min. Then, T4 DNA ligase (1.5 U μl^−1^; Fermentas) and T4 RNA ligase 2 (22 ng μl^−1^) were added and the ligation was performed in the thermomixer at 16 °C overnight. Template DNA was removed by the addition of 1.5 U μl^−1^ DNase I (Fermentas), followed by 1 h of incubation at 37 °C. The tRNAs were purified from ligation mixtures by denaturing polyacrylamide gel electrophoresis, excised and eluted from the gel, and precipitated with ethanol. Concentrations were calculated from absorption at 254 nm, determined using a Nanodrop ND-1000 spectrometer (Thermo Scientific, Waltham, MA, USA).

### *In vitro* aminoacylation reactions

*In vitro* aminoacylation was performed using the synthesized murine-tRNA^Asp^, which was either methylated or unmethylated at the C38 position. For the *in vitro* aminoacylation, 100 nm to 1 μm tRNA substrate (as indicated) was incubated with 50 nm of the recombinant AspRS in aminoacylation buffer (50 mm PIPES pH 7.0, 30 mm KCl, 10 mm MgCl_2_, 1 mm DTT, and 2 mm ATP) containing 1 μm [^3^H]-aspartate (MP Biomedicals, Eschwege, Germany; 9.25 MBq) at 37 °C. The reaction progress was monitored by removing 5-μl aliquots of the reaction mixture at specific time points and precipitating them in 500 μl of 5% trichloroacetic acid (TCA). Then, the samples were spotted on a DE81 filter paper, washed with 5% TCA followed by an ethanol wash, and the radioactivity was counted in a Hidex 300 SL liquid scintillation counter (Hidex, Turku, Finland) using Rotiszint eco plus scintillation liquid (Roth, Karlsruhe, Germany). The reaction progress curves were fitted by linear regression analysis and the reaction rates were used to calculate the *V*_max_/*K*_m_ by least-squares fitting of the data to the Michaelis–Menten equation.

### Cell lines, culture conditions, and transfection

Wild-type and Dnmt2 KO MEF cells were prepared as described [13] and were grown in standard Dulbecco's modified Eagle's media containing 10% fetal bovine serum and supplemented with 2 mm Glutamate in a 5% CO_2_ incubator. The cells were grown to 90% confluence and split into a six-well plate prepared with cover slides at a seeding density of 1×10^6^ cells per well. Afterwards, the cells were grown for another 24 h in Dulbecco's modified Eagle's medium to reach ~50% confluence. Then, the MEF cells were transfected with a fluorescent reporter construct (yellow fluorescent protein (YFP) and cyan fluorescent protein (CFP)) with and without poly-Asp_6_-coding sequence at their N-terminal side using Xfect mESC Transfection Reagent (Clontech, Mountain View, CA, USA) following the instructions of the supplier. The medium was exchanged 3 h after transfection.

### Fluorescence microscopy

After 48 h, the cells transfected with reporter constructs were washed three times with 2 ml of phosphate-buffered saline, fixed using 3.7% formaldehyde solution for 10 min and washed three more times with phosphate-buffered saline. After this, the cover slides were carefully removed from the plate and mounted on a base slide spotted with 50 μl of Mowiol (48 mg ml^−1^ Mowiol dissolved in 0.2 m Tris/HCl pH 8.5 containing 0.12 g ml^−1^ glycerol). The slides were kept in the dark for 2–3 h and subsequently sealed to avoid drying. Fluorescent images were taken using a Zeiss Laser Scanning Microscope 510 (Carl Zeiss, Jena, Germany) with ×20 magnification using specific filters for CFP (430 nm excitation and 475 nm emission readout) and YFP (510 nm excitation and 530 nm emission readout). The fluorescent intensities from more than 150 individual cells were quantified using the ImageJ analysis tool (rsb.info.nih.gov/ij/).

### Determination of cellular aminoacylation levels of tRNA^Asp^

For the measurement of aminoacylation level of tRNA^Asp^ in cells, the tRNA was isolated from early passage (P10) wild-type and Dnmt2 KO MEF cells under a mild acidic condition, which preserves the aminoacylation [31, 32]. For this, the cells were grown in a T75 flask until they reached 90% confluence, trypsinized, and collected in 500 μl of 0.3 m sodium acetate pH 4.5 and 10 mm EDTA followed by addition of an equal volume of sodium acetate-saturated phenol/chloroform pH 4.5. The cell pellet was vortexed three times for 30 s with 30-s pauses on ice. The supernatant was collected by centrifugation at 18 600 *g* for 30 min at 4 °C. The RNA was then precipitated by addition of 2.5 volumes of ethanol and resuspended in 10 mm sodium acetate pH 4.5 and 1 mm EDTA. The isolated RNA containing tRNA was then divided into two fractions; one of these parts was treated with alkaline buffer (100 mm Tris-Cl pH 8.0 and 100 mm NaOH) at 37 °C to deacylate the tRNAs [33]. The tRNA was precipitated and resuspended in RNase-free water. The total concentration of the alkaline-treated and untreated tRNA was calculated from absorbance at 254 nm as determined with a Nanodrop ND-1000 spectrometer and confirmed by gel analysis. To determine the aminoacylation level of the tRNA, an incorporation assay was used. For the assay, equal amounts of the tRNA fractions were incubated with 50 nm AspRS in the presence of 1 μm [^3^H]-aspartate and aminoacylation buffer at 37 °C. The reaction was stopped after 90 min by TCA precipitation. The samples were spotted on DE81 filter discs and washed with 5% TCA and absolute ethanol. Following this, the DE81 discs were air dried and the incorporated radioactivity was counted using Hidex 300 SL liquid scintillation counter (Hidex) using Rotiszint eco plus. The aminoacylation of the tRNA^Asp^ was quantified from the ratio of [^3^H]-labelled tRNA^Asp^ in the samples isolated from wild-type and Dnmt2 KO cells. Counts from the corresponding deacylated samples were used to normalize the input of tRNA^Asp^ in both samples. In this acceptor assay method, the counts obtained from the acylated samples are inversely proportional to their charging levels.

### Western blotting

Early passage (P10) wild-type and Dnmt2 KO MEF cells were grown till 90% confluence and the cell pellet was collected after trypsination. Protein extracts were prepared by cell lysis using RIPA buffer. The protein amount was determined by absorbance at 280 nm using a Nanodrop ND-1000 spectrometer and equal amounts were separated on a 15% SDS gel. After transferring the proteins to a nitrocellulose membrane, specific proteins were detected with antibodies against poly-aspartate-containing proteins. The candidate proteins and the antibodies used against them are listed in Supplementary Table S3. Antibodies were used at dilutions specified by the supplier for western blotting. After washing three times with Tween tris buffered saline (TTBS) buffer, the blots for Taf9, protein-SET, Daxx, and NPM were incubated with anti-rabbit (GE Healthcare NA934 in 1:5 000) secondary antibody, whereas blots for FGFR1, Ezh2, and TFDP-1 were incubated with anti-mouse (NA931 in 1:5 000, GE Healthcare, Frankfurt, Germany) secondary antibody for 2 h at room temperature and developed using enhanced chemiluminescence western blotting solution (Thermo Scientific, Braunschweig, Germany). Images were captured on an X-ray film, scanned, and analysed using ImageJ (rsb.info.nih.gov/ij/). For loading control, either Ponceau stain or rabbit anti-β-actin IgG (AbCam ab8227 in 1:5 000) followed by goat anti-rabbit IgG conjugated to horseradish peroxidase (GE Healthcare) was used.

### *In vivo* protein degradation analysis

To study protein degradation in wild-type and Dnmt2 KO MEFs, cells were grown in a six-well plate in Dulbecco's modified Eagle's medium to 90% confluence and cycloheximide was added to a final concentration of 50 μg ml^−1^. This stops ongoing protein synthesis such that protein degradation rates can be observed. The cells were incubated for six more hours after cycloheximide addition. At specific time points cells from a single well for each wild-type and Dnmt2 KO were trypsinized and lysed in RIPA buffer (50 mm Tris pH 8.0, 150 mm NaCl, 1% NP40, 0.25% sodium deoxycholate, and 1 mm phenylmethylsulphonyl fluoride). Equal amounts of protein were separated on a 15% SDS-polyacrylamide gel electrophoresis gel and transferred to a nitrocellulose membrane. The blots were blocked in 5% milk overnight at 4 °C and incubated with corresponding primary antibodies as specified above or rabbit anti-β-actin IgG (AbCam ab8227 in 1:5 000) antibodies followed by goat anti-rabbit IgG conjugated to horseradish peroxidase (GE Healthcare). The blots were developed using enhanced chemiluminescence western blotting solution (Thermo Scientific). Images were captured on an X-ray film, scanned, and analysed using ImageJ.

## Figures and Tables

**Table 1:** Gene ontology (GO) classification of human proteins with poly-Asp sequence motifs

*Description*	*No. of proteins*	P*-value*
*Proteins containing a D4 sequence*		
Total no. of proteins	306	
Mapped with GO classification	185	
Nuclear proteins	86	6.76×10^−10^
Regulation of gene expression	51	6.71×10^−5^
Nucleic acid binding	52	1.80×10^−3^
		
*Proteins containing a D5 sequence*		
Total no. of proteins	118	
Mapped with GO classification	68	
Nuclear proteins	34	4.66×10^−5^
Nucleic acid binding	22	2.86×10^−2^
Chromosome organization	9	3.50×10^−3^
		
*Proteins containing a D6 sequence*		
Total no. of proteins	50	
Mapped with GO classification	29	
Nuclear proteins	16	2.00×10^−3^
Transcription regulator	9	8.80×10^−3^
Gene expression	13	1.54×10^−2^

A major proportion of proteins are associated with nuclear function and gene expression (prepared using Gene set analysis toolkit V2 http://bioinfo.vanderbilt.edu/webgestalt/).
