# Supplementary material for: Cytosine methylation of tRNA-Asp by DNMT2 has a role in translation of proteins containing poly-Asp sequences
Source: Cell Discov. 2015 Jun 9;1:15010–. doi: 10.1038/celldisc.2015.10 (PMC4860778; doi:10.1038/celldisc.2015.10)
Supplement: Supplementary Information [file celldisc201510-s1.pdf]

Cytosine methylation of tRNA-Asp by DNMT2 has a role in translation of proteins containing poly-Asp sequences

Raghuvaran Shanmugam, Jacob Fierer, Steffen Kaiser, Mark Helm, Tomasz P. Jurkowski, & Albert Jeltsch

Supplemental information

Suppl. Figures 1-5

Suppl. Tables 1-3

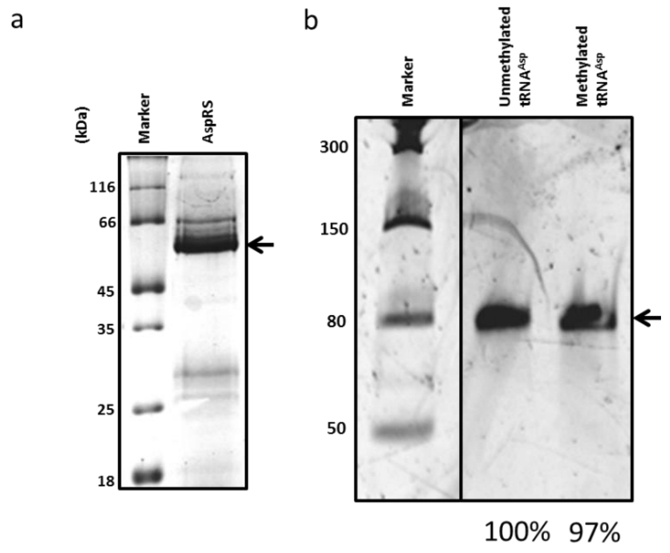

Suppl. Figure 1: Exemplary gels showing the tRNA substrates and AspRS enzymes used in this study. a) Coomassie-stained protein gel showing the purification of the AspRS enzyme used in the study. b) Image of a Gel Red-stained polyacrylamide gel showing the corresponding amounts of substrate tRNAs used in the kinetic analysis.

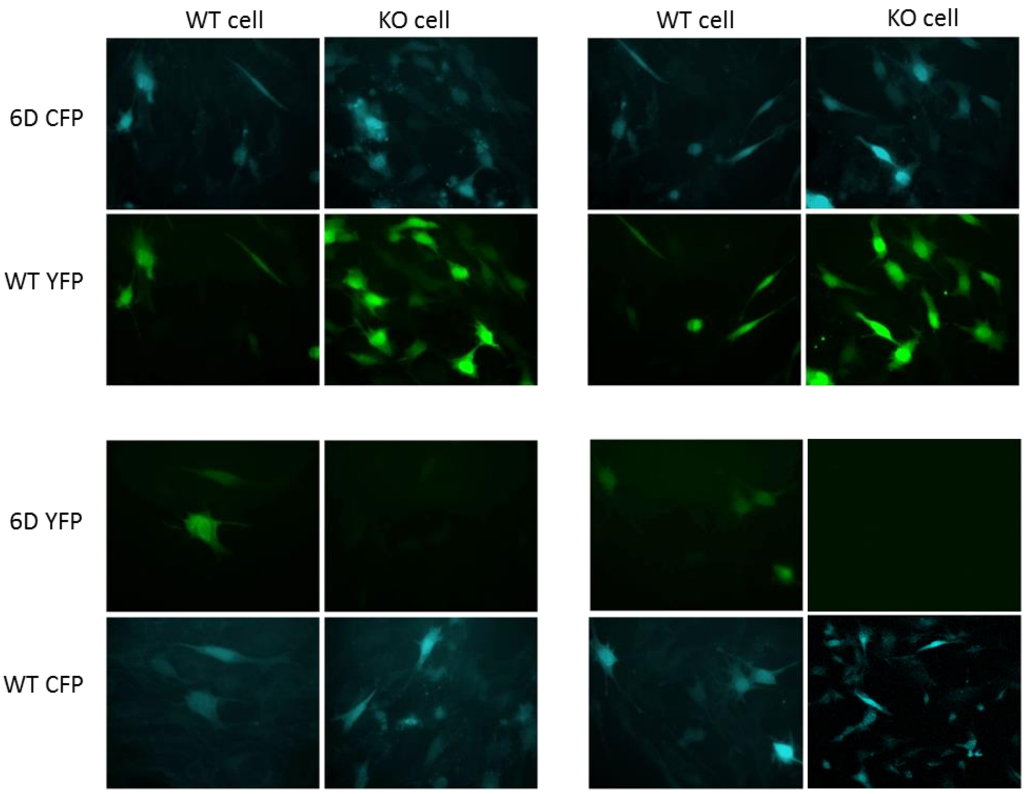

Suppl. Figure 2: Additional example pictures of wild type and Dnmt2 KO cells co-transfected with 6D YFP/CFP or vice versa (cf. Figure 3). In Dnmt2 KO cells the 6D YFP/6D CFP proteins showed a reduced synthesis compared to the CFP/YFP protein respectively. The images were taken 48 hours after transfection and the cells were fixed by formaldehyde.

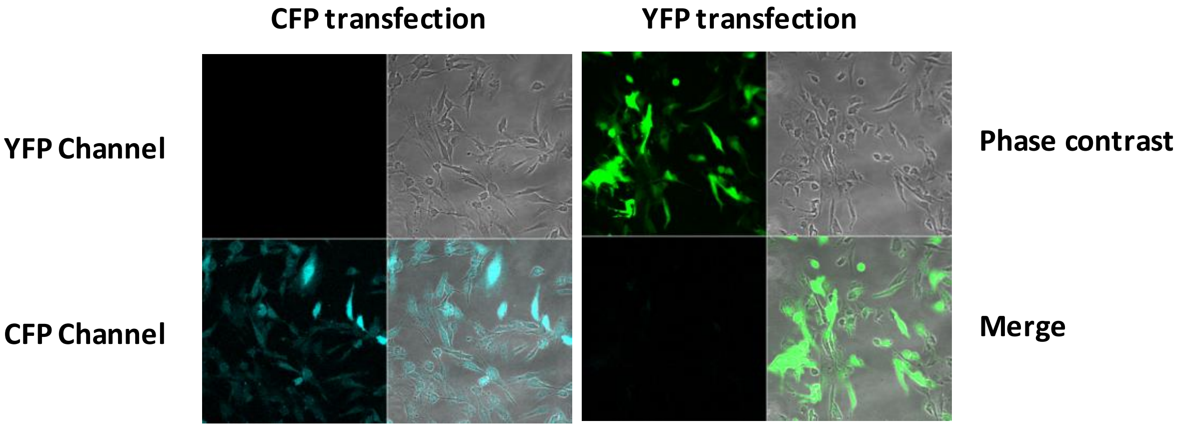

Suppl. Figure 3: Images of cells transfected only with YFP or CFP in the different fluorescent channels showing the absence of crosstalk between the fluorophores (cf. Fig. 3).

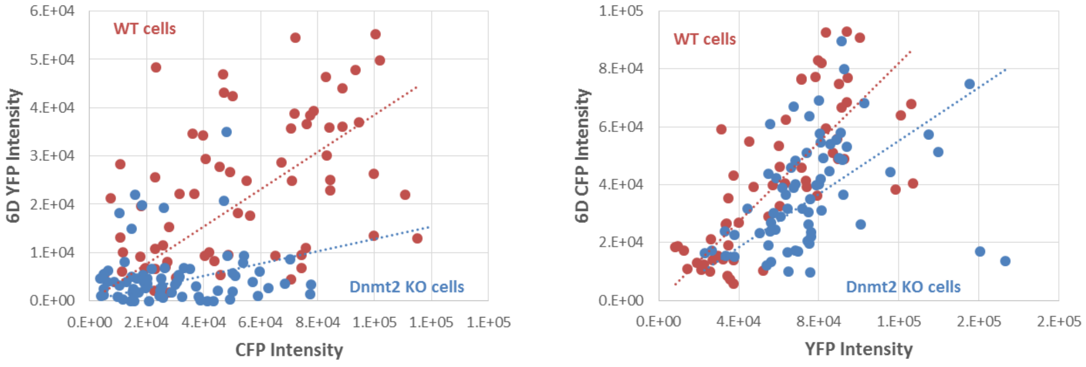

Suppl. Figure 4: Quantitation of the YFP and CFP fluorescence in many individual wild type and Dnmt2 KO cells co-transfected with 6D YFP/CFP or vice versa. Approximately 150 cells were analysed for each experiment. The lines show linear regressions and are only shown for comparison. Compilations of these data are shown in Figure 4.

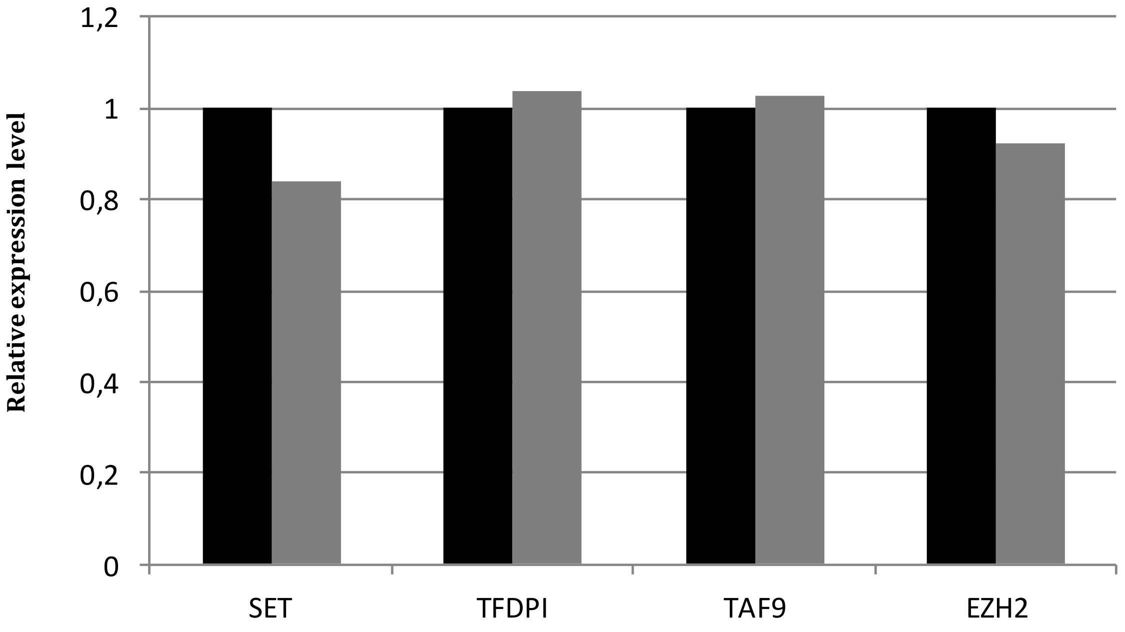

Suppl. Figure 5: Gene expression of the proteins containing poly Asp-runs analysed in this study. The mRNA levels of the candidate genes was compared between the MEFs wild type (GSM906315) and MEFs Dnmt2 KO (GSM906316) cells based on the reported data from the whole transcriptome analysis (GEO accession: GSE36918) (taken from Tuorto, et al. (2012) *Nature structural & molecular biology* 2012; **19**:900-905). The expression of candidate gene was unaffected for all the proteins except protein-SET that showed a moderate reduction in the Dnmt2 KO cells. These experiment were conducted in the same cell line as used in the current study.

Protein	Amino acid sequence
Taf9	MESGKMASPKSMPKDAQMMAQILKDMGITEYEPRVINQMLEFAFRYVTTILDDAKIY<br>SSHAKKATVDADDVRLAIQCRADQSFTSPPPRDFLLDIARQRNQTPLPLIKPYSGPRLPP<br>DRYCLTAPNYRLKSLQKKAPAPAGRITVPRLSVGSVSSRPSTPTLGTPTPQTMSVSTKVG<br>TPMSLTGQRFTVQMPASQSPAVKASIPATSTVQNVLINPSLIGSKNILITTNMVSQNTA<br>ESANALKRKREDDDDDDDDDDDDDYDNM
Ezh2	MGQTGKKSEKGPVCWRKRVKSEYMRLRQLKRFRRADEVKTMFSSNRQKILERTETLN<br>QEWKQRRIQPVHIMTSVSSLRGTRECSVTSDLDFPAQVIPLKTLNAVASVPIMYSWSPL<br>QQNFMVEDETVLHNIPYMGDEVLDQDGTFIEELIKNYDGKVHGDRECGFINDEIFVEL<br>VNALGQYNDDDDDDDGDDPDEREEKQKDLEDNRDDKETCPPRKFPADKIFEAISSMF<br>PDKGTAEELKEKYKELTEQQLPGALPPECTPNIDGPNAKSVQREQSLHSFHTLFCRRCFK<br>YDCFLHPFHATPNTYKRKNTETALDNKPCGPQCYQHLEGAKEFAAALTAERIKTPPKRP<br>GGRRRGRLPNNSSRPSTPTISVLESKDTDSDREAGTETGGENNDKEEEEKKDETSSSSEA<br>NSRCQTPIKMKPNIEPPENVEWSGAEASMFRVLIGTYYDNFCAIARLIGTKTCRQVYEF<br>RVKESSIIAPVPTEDVDTPPRKKKRKHRLWAAHCRKIQLKKDGSSNHVYNYQPCDHPR<br>QPCDSSCPCVIAQNFCEKFCQCSSECQNRFPGCRCKAQCNTKQCPCYLAVRECDPDLC<br>LTCGAADHWDSKNVSCKNCSIQRGSKKHLLLAPSDVAGWGIFIKDPVQKNEFISEYCGE<br>IISQDEADRRGKVYDKYMCSFLFNLNNDFVVDATRKGNKIRFANHSVNPNCYAKVMM<br>VNGDHRIGIFAKRAIQTGEELFFDYRYSQADALKYVGIEREMEIP
Tfdp1	MAKDASLIEANGELKVFIDQNLSPGKGVVSLVAVHPSTVNTLGKQLLPKTFGQSNVNIT<br>QQVVIGTPQRPAASNTIVVGSPHTPNTHFVSQNQTSDSSPWSAGKRNRKGEKNGKGL<br>RHFSMKVCEKVQRKGTTSYNEVADELVAEFSAADNHILPNESAYDQKNIRRRVYDALN<br>VLMAMNIISKEKKEIKWIGLPTNSAQECQNLEVERQRRLERIKQKQSQLQELILQQIAFK<br>NLVQRNRQAEQQARRPPPPNSVIHLPFIIVNTSRKTVIDCSISNDKFEYLFNFDNTFEIHD<br>DIEVLKRMGMACGLESGNCSAEDLKVARSLVPKALEPYVTEMAQGSIGGVFVTTTGST<br>SNGTRLSASDLSNGADGMLATSSNGSQYSGSRVETPVSYVGEDDDDDDDFNENDEED
Protein- SET	MAPKRQSAILPQPKKPRPAAAPKLEDKSASPGLPKGEKEQQEAIEHIDEVQNEIDRLNE<br>QASEEILKVEQKYNKLRQPFFQKRSELIAKIPNFWVTTFVNHPQVSALLGEEDEEALHYL<br>TRVEVTEFEDIKSGYRIDFYFDENPYFENKVLSKEFHLNESGDPSSKSTEIKWKSGKDLTK<br>RSSQTQNKASRKRQHEEPESFFTWFTDHSDAGADELGEVIKDDIWPNPLQYYLVPDM<br>DDEEGEAEDDDDDDEEEEGLEDIDEEGDEDEGEEDDDEDEGEEGEEDEGEDD
NPM	MEDSMDMDMSPLRPQNYLFGCELKADKDYHFKVDNDENEHQLSLRTVSLGAGAKDE<br>LHIVEAEAMNYEGSPIKVTLATLKMSVQPTVSLGGFEITPPVVLRLKCGSGPVHISGQHL<br>VAVEEDAESEDEDEEDVKLLGMSGKRSAPGGGNKVPQKKVKLDEDDEDDDEDDEDD<br>EDDDDDDFDEEETEEKVPVKKSVRDTPAKNAQKSNQNGKDLKPSTPRSKGQESFKKQE<br>KTPKTPKGPSSVEDIKAKMQASIEKGGSLPKVEAKFINYVKNCFRMTDQEAIQDLWQW<br>RKSL
DAXX	MATDDSIIVLDDDDEDEAAAQPGPSNLPPNPASTGPGPGLSQQATGLSEPRVDGGSS<br>NSGSRKCYKLDNEKLFEEFLELCKTETSDHPEVVPFLHKLQQRAQSVFLASAEFCNILSRV<br>LARSRKRPAKIYVVYINELCTVLKAHSIKKKLNLAPAASTTSEASGPNPPTEPPSDLTNTENT<br>ASEASRTRGSRRQIQRLEQLLALYVAEIRRLQEKELDLSELDDPDSSYLQEARLKRKLIRLF<br>GRLCELKDCSSLTGRVIEQRIPYRGTRYPEVNRRIERLINKPGLDTFPDYGDVLRAVEKAA<br>TRHSLGLPRQQLQLLAQDAFRDVGVRLQERRHLDLIYNFGCHLTDDYRPGVDPALSDP<br>TLARRLRENRTLAMNRLDEVISKYAMMQDKTEEGERQKRRARLLGTAPQPSDPPQAS

	SESGEGPSGMASQECPTTSKAETDDDDDDDDDDDEDNEESEEEEEEEEEEKEATEDED EDLEQLQEDQGGDEEEEGGDNEGNESPTSPSDFFHRRNSEPAEGLRTPEGQQKRGLTE TPASPPGASLDPPSTDAESSGEQLLEPLLGDESPVSQLAELEMEALPEERDISSPRKKSED SLPTILENGAAVVTSTSVNGRVSSHTWRDASPPSKRFRKEKKQLGSGLLGNSYIKEPMA QQDSGQNTSVQPMPSPPLASVASVADSSTRVDSPSHELVTSSLCSPSPSLLLQTPQAQS LRQCIYKTSVATQCDPEEIIVLSDSD

Suppl. Table 1: Sequences of the poly-Asp stretch containing proteins investigated in this work. All protein parameters are listed in Supplemental Table 3. FGFR1 could not be studied due to failure of the antibody.

Construct name	5‘ fragment		3‘ fragment	
**$tRNA^{Asp}$**	MH 565	UCCUCGUUAGUAUAG UGGUGAGUAUCCCCG CCU	MH 566	GUCACGCGGGAGACCGGGGUUCG AUUCCCCGACGGGGAGCCA
**$tRNA^{Asp}$ m^5C38**	MH 565	UCCUCGUUAGUAUAG UGGUGAGUAUCCCCG CCU	MH 606	GUCAm^5CGCGGGAGACCGGGGUU CGAUUCCCCGACGGGGAGCCA
**Splint**	MH 570	dGdGdAdAdTdCdGdAdAdCdCdCdCdGdGdTdCdTdCdCdCdGdCdGdTdGd AdCdAdGdGdCdG dGdGdGdAdTdAdCdTdCdAdCdCdAdCdTdAdTdAdC			

Suppl. Table 2: Sequences of mouse $tRNA^{Asp}$ fragments and the DNA splint used in the synthesis of unmethylated and C38 methylated $tRNA^{Asp}$ (methyl cytosine is indicated in red)

**Protein**	**Short name**	**SwissProt ID**	**Antibody Cat.No**
Phosphatase 2A inhibitor I2PP2A	Protein SET	Q9EQU5	PA5-21756 (Thermo scientific)
Transcription factor Dp-1	TFDP1	Q08639	MA5-11268 (Thermo scientific)
Transcription initiation factor TFIID subunit 9	TAF9	Q8VI33	10544-1-AP (protein tech)
Histone-lysine N-methyltransferase	EZH2	Q61188	MA5-15101 (Thermo scientific)
Death domain-associated protein 6	DAXX	O35613	PA5-19885 (Thermo scientific)
Nucleophosmin	NPM	Q61937	10306-1-AP (protein tech)
Basic fibroblast growth factor receptor 1	FGFR1	P16092	MA1-26256 (Thermo scientific)

Suppl. Table 3: Compilation of the candidate proteins selected for our analysis and the respective antibodies used.
